# Predicting Factors of Clinical Outcomes in Patients Hospitalized after Esophageal Foreign Body or Caustic Injuries: The Experience of a Tertiary Center

**DOI:** 10.3390/diagnostics13213304

**Published:** 2023-10-25

**Authors:** Tiago Ribeiro, Miguel Mascarenhas Saraiva, João Afonso, Lorenzo Brozzi, Guilherme Macedo

**Affiliations:** 1 Department of Gastroenterology, Centro Hospitalar Universitário de São João, 4200-427 Porto, Portugal; tiagofcribeiro@outlook.com (T.R.); guilhermemacedo59@gmail.com (G.M.); 2 WGO Gastroenterology and Hepatology Training Center, 4200-319 Porto, Portugal; 3 Department of Medicine, Faculdade de Medicina da Universidade do Porto, 4200-319 Porto, Portugal; 4 Gastroenterology and Digestive Endoscopy Unit, Pancreas Institute, Department of Medicine, G.B. Rossi University Hospital, 37134 Verona, Italy; lorenzo.brozzi89@gmail.com

**Keywords:** foreign bodies, caustics, endoscopy, computed tomography, length of stay

## Abstract

Ingestion of foreign bodies (IFB) and ingestion of caustic agents are frequent non-hemorrhagic causes of endoscopic urgencies, with the potential for severe complications. This study aimed to evaluate the predicting factors of the clinical outcomes of patients hospitalized as a result of IFB or ingestion of caustics (IC). This was a retrospective single-center study of patients admitted for IFB or IC between 2000 and 2019 at a tertiary center. Demographic and clinical data, as well as preliminary exams, were evaluated. Also, variables of the clinical outcomes, including the length of stay (LS) and other inpatient complications, were assessed. Sixty-six patients were included (44 IFB and 22 IC). The median LS was 7 days, with no differences between the groups (*p* = 0.07). The values of C-reactive protein (CRP) upon admission correlated with the LS in the IFB group (*p* < 0.01) but not with that of those admitted after IC. In the IFB patients, a diagnosis of perforation on both an endoscopy (*p* = 0.02) and CT scan (*p* < 0.01) was correlated with the LS. The Zargar classification was not correlated with the LS in the IC patients (*p* = 0.36). However, it was correlated with antibiotics, nosocomial pneumonia and an increased need for intensive care treatment. CT assessment of the severity of the caustic lesions did not correlate with the LS. In patients admitted for IFB, CRP values may help stratify the probability of complications. In patients admitted due to IC, the Zargar classification may help to predict inpatient complications, but it does not correlate with the LS.

## 1. Introduction

Foreign body ingestion, including food bolus impaction, and caustic injuries are common endoscopic urgencies. Both entities have the potential for serious complications and frequently occur in vulnerable patients, notably children, the elderly and patients with cognitive impairment or psychiatric disease [1].

Foreign body ingestion most frequently occurs accidentally in young children [1,2]. In adults, most cases are accidental, and the predominant foreign bodies vary between different zones of the globe. In the Western Hemisphere, food impaction predominates, whereas the ingestion of bones occurs more frequently in Asia [3,4]. Depending on the series, concomitant esophageal disease is found in 5–31% of patients [2,4,5,6,7,8,9]. These patients are primarily affected by food impaction [10]. Most FBs eventually pass spontaneously. Endoscopic retrieval is the current mainstay for treatment and is associated with high rates of success [7]. Nevertheless, it is only required in 10–20% of cases [1]. Most studies report complication rates ranging between 2 and 20%, with most complications successfully managed via medical or endoscopic strategies [2,3,4,7,8,9,10,11,12,13].

Caustic injury more frequently refers to tissue damage after either acid or alkali ingestion. This is a common public health problem, particularly in lower-income countries [14]. IC may occur accidentally or intentionally. Accidental ingestion predominates in children and often presents with milder lesions. Intentional ingestion is more frequent in adults, particularly those with histories of psychiatric disease, and is associated with increased lesion severity [14,15]. Endoscopic evaluation has been the mainstay for the assessment of the severity and extension of the caustic damage and, therefore, the selection of the therapeutic approach. Endoscopic severity grading using the Zargar classification has been proven to correlate with the disease severity, acute complications, mortality and late complications, such as stricture formation [16,17]. Nevertheless, this view has been challenged by evidence suggesting the superiority of the CT scan for selecting candidates for surgical versus non-operative treatment, with improved survival outcomes [18,19].

The integration of clinical, laboratory, endoscopic and imaging findings is essential for the management of patients with IFB or IC. This study aimed to evaluate the predicting factors of the clinical outcomes of patients hospitalized for IFB or IC in a tertiary referral hospital.

## 2. Materials and Methods

### 2.1. Demographic and Clinical Data

The medical records of all consecutive patients admitted as inpatients for IFB or IC in a tertiary university hospital (Centro Hospitalar Universitário de São João) were reviewed. A total of 66 cases (44 IFB and 22 IC), in a period between 2000 and 2019, were ultimately analyzed. This study was approved by the Institutional Review Board at Centro Hospitalar Universitário de São João. For each patient, the type of injury was labeled as either IFB or IC. Demographic parameters, such as age and gender, were included. Clinical history parameters, including the comorbidity burden, as assessed using the Charlson comorbidity index (CCI), as well as a history of psychiatric disease and the circumstances leading to the event (voluntary versus unvoluntary ingestion), were registered. Symptoms at presentation were registered and included dysphagia or odynophagia, thoracic or abdominal pain, vomiting, drooling, hematemesis, melena and dyspnea. Furthermore, admission lab values, including hemoglobin (Hb), white blood cell (WBC) count and C-reactive protein (CRP), were noted.

### 2.2. Endoscopic and Imaging Data

Endoscopy was performed in 61 of the 66 patients (92%). The time between presentation and endoscopy was documented in hours and categorized as occurring <24 h or ≥24 h after presentation. Moreover, data regarding the site of injury and type of lesion were collected. In the case of IFB, the occurrence of mucosal ulceration or organ perforation was registered. Mucosal damage due to IC was assessed using the endoscopic classification of Zargar [20]: 0—no lesions; 1—mucosal edema and hyperemia; 2A—superficial ulcers, exudates, whitish membranes, friability, blisters and bleeding; 2B—grade 2A plus deep focal or circumferential ulcers; 3A—small scattered areas of focal necrosis; 3B—extensive necrosis. For further analysis, these lesions were categorized as mild IC (1–2A) and moderate-to-severe IC (≥2B).

CT scans were performed on 48 patients (73%). Reports of CT scans were reviewed for alterations due to IFB or IC (namely, parietal edema, with or without soft-tissue involvement and periesophageal collections, or perforation). A CT scan severity index was then calculated for patients with IC, as suggested by Ryu et al. [21]: I—no alterations on CT scan; II—edematous esophageal wall thickening without soft-tissue involvement*;* III—edematous esophageal wall thickening with periesophageal soft-tissue infiltration; IV—edematous wall thickening with periesophageal soft-tissue infiltration and blurring of tissue interface or periesophageal fluid collection.

### 2.3. Study Outcomes

The associations between the demographic, clinical, endoscopic and imaging variables and inpatient outcomes were evaluated. The primary outcome variable of this study was the LS. Other outcome variables included the need for treatment in an intensive care unit (ICU), vasopressor support and complications such as hospital-acquired pneumonia, the need for antibiotic treatment, hollow viscera perforation, peritonitis or mediastinitis.

### 2.4. Statistical Analysis

Categorical variables are expressed in numbers and percentages. Comparison between categorical variables, as well as their correlations, were performed using Fisher’s exact test and Pearson’s φ, respectively. Normally distributed continuous variables are expressed with means and standard deviation, whereas non-normally distributed variables are described using the median and interquartile range. Continuous variables were compared using the Mann–Whitney U test or Kruskal–Wallis test, as appropriate. Correlations were assessed using Spearman’s correlation coefficient (*ρ*). A *p*-value < 0.05 was required for statistical significance.

## 3. Results

### 3.1. Clinical and Demographic Data

A total of 66 patients (64% male patients) were hospitalized after IFB (*n =* 44) or IC (*n =* 22). The mean age of all included patients was 54 years, with no significant difference between those with caustic injury or IFB (*p* = 0.25). The CCI had a median value of 3, with no significant difference between the IFB and IC patients (*p* = 0.109). A previous diagnosis of psychiatric disease was present in 26 (39%) patients and occurred more frequently in those hospitalized for IC (*p* < 0.0001). Of these patients, 25 had a diagnosis for review. The most frequently diagnosed conditions were depression (*n* = 9) and alcoholism (*n* = 7). Moreover, intentional ingestion more frequently occurred in those admitted after IC than IFB (*p* < 0.0001). The existence of a previous diagnosis of psychiatric disease strongly correlated with the occurrence of self-inflicted lesions (Pearson’s φ 0.742, *p* < 0.0001). Table 1 summarizes the demographic analysis and patients’ characteristics of both groups.

Foreign bodies were found and reported in 24 IFB cases and included fish bones (*n* = 9), food boluses (*n* = 6), meat bones (*n* = 4), dental protheses (*n* = 3), a razor blade (*n* = 1) and a glass fragment (*n* = 1). Alkali ingestion was responsible for the majority of IC admissions (*n* = 15), followed by bleaches (*n* = 5) and acid ingestion (*n* = 2).

Overall, there were no significative differences in the frequency of the presenting symptoms between the two groups (Table 1). Remarkably, four IFB patients (10%) and two IC patients (12%) were asymptomatic. Odynophagia and dysphagia were the most frequent symptoms, affecting almost 72% of all patients, with no differences between the two subsets of patients (*p* = 0.16). IC patients showed a tendency to more frequently present with hematemesis compared to IFB patients (35% versus 13%), although the difference did not reach statistical significance (*p* = 0.07). Blood tests performed upon admission revealed no significant differences in the mean levels of hemoglobin or the white blood cell count, or in the median C-Reactive protein (CRP) values (Table 1).

### 3.2. Endoscopy Findings

A total of 61 patients (92%) underwent upper endoscopy, 41 after IFB and 20 after IC. The technique was performed within the first 24 h after presentation in most patients (77%). No differences in the timing for the endoscopy were observed between the two groups of patients (*p* = 0.517). In those admitted after IFB, the foreign body was found in 24 patients (55%). Table 2 describes the results of the endoscopic evaluations of both groups.

Most lesions in the IFB group were located in the upper and middle thirds of the esophagus (27% and 44%, respectively). The most frequently reported foreign body lesion was ulceration of the esophageal mucosa (42%), followed by perforation (29%) and hematoma (21%). No lesions were found in almost 30% of these patients. Conversely, most patients admitted after ingestion of caustics had pan-esophageal involvement (61%). The severity of lesions occurring after caustic ingestion was assessed using the Zargar classification. Hyperemia and mucosal edema (grade 1) were the most common findings. Nevertheless, more than half of the IC patients had moderate-to-severe lesions.

### 3.3. CT Scan Findings

A CT scan was performed on 48 patients (73%): 34 after IFB and 14 after IC. The imaging findings of these patients are summarized in Table 3. The most frequent finding in those hospitalized due to IFB was perforation, which was detected in approximately 53% of all CT scans. Perforations on CT scans were more frequently detected in IFB patients than those admitted after IC (*p* = 0.02). In the latter group, edema of the esophageal wall was the most common finding, and those with demarcated soft-tissue involvement occurred more frequently compared to IFB patients (*p* = 0.02). Additionally, more than half of the IC patients had periesophageal soft-tissue involvement (CT scan severity index III–IV). Edema with or without soft-tissue involvement was present in half of the IFB patients. The existence of periesophageal fluid collections on the CT scans of IFB patients (21%) was limited to those with concomitant perforation.

Among all patients, the median length of stay was 7 days (Table 4). Those admitted for caustic injury demonstrated a tendency towards a longer stay, although this difference was insufficient to reach statistical significance (9 versus 6 days, *p* = 0.07). No difference existed between the groups regarding the need for treatment in an intensive care unit, vasopressor support, the need for antibiotics or complications such as pneumonia and respiratory failure. The cases of esophageal injury complicated with mediastinitis occurred in similar percentages in both sets of patients and, overall, affected almost 20% of all patients. Perforation, diagnosed either by endoscopy or CT, was more frequent after IFB (46% versus 9%, *p* = 0.01). Most complications in both groups received medical treatment, with no significant differences between the IFB and IC patients (*p* = 0.283). Surgical treatment was required in 12 IFB patients, of whom 10 had esophageal perforation. The remaining patients with perforation received either medical treatment alone (*n* = 8) or an endoscopic stent (*n* = 2). Mediastinitis in IFB patients (*n* = 9) was present in those with perforation. Surgical treatment was the option for five of these patients. None of the 66 cases of IFB or IC culminated in the death of the patient during hospitalization.

Several clinical, laboratorial, imaging and endoscopic findings were evaluated in order to establish eventual correlations with the clinical outcomes of patients admitted for IFB or IC (Table 5). Age and the comorbidity burden, measured via the CCI, did not correlate with the length of hospitalization globally, nor for each group individually. Voluntary ingestion was more common for caustics than for foreign bodies. However, there were no differences in the duration of the hospital stay according to the motivation for IFB or IC, either globally (*p* = 0.56) or for each group individually (*p* = 0.13 and *p* = 0.76, respectively). Regarding the lab values, no correlation existed between the levels of hemoglobin and the length of hospitalization (*p* = 0.97 overall; *p* = 0.93 and *p* = 0.82 for IFB and IC, respectively). Similarly, no association was found between the WBC count and length of stay. However, the CRP values were significantly correlated with this outcome in those admitted for IFB (*p* < 0.01). Such a correlation was not evident in patients admitted for IC (*p* = 0.44). Moreover, IFB patients with CRP levels > 50 mg/L more frequently required surgical treatment for complications (*p* = 0.004).

The timing of the endoscopy did not correlate with the LS in either group of patients. Also, delay in endoscopy (≥24 h) was neither correlated to the latter nor to the other inpatient measures of outcome (such as the need for ICU, antibiotics, respiratory failure, pneumonia, perforation, peritonitis, mediastinitis or the need for surgery) in both subgroups of patients. The presence of endoscopic lesions in patients hospitalized for IFB appeared to have an influence on the outcome. These patients had longer hospital stays compared to those without endoscopic lesions (*p* = 0.03). However, such a difference appeared to be restricted to those patients with endoscopic detection of perforation (*p* = 0.02). In fact, the existence of endoscopic lesions in the IFB patients was moderately but significantly correlated with longer hospitalizations (Spearman’s *ρ* 0.43, *p* = 0.01). Conversely, no correlation was found between the severity of the caustic lesions (assessed by the Zargar classification) and length of hospitalization (*p* = 0.36). Nevertheless, the presence of moderate-to-severe lesions was found to be associated with an increased need for ICU treatment (*p* = 0.02), the use of antibiotics (*p* = 0.01) and the incidence of respiratory complications, such as nosocomial pneumonia (*p* = 0.03).

Among the findings of the CT scans, the IFB patients with findings of periesophageal collections or perforation had longer hospitalizations (*p* = 0.04 and *p* < 0.01, respectively). In fact, in this subset of patients (but not with the IC patients), such findings correlate with more prolonged stays (*ρ* 0.36, *p* = 0.04 for periesophageal collections and *ρ* 0.63, *p* < 0.01 for perforation). Conversely, the calculated CT scan severity index had no established correlation with the length of hospitalization in the patients with caustic injury (*p* = 0.21).

## 4. Discussion

The ingestion of foreign bodies is a common cause for emergency endoscopy. Data from North America estimate 120,000 annual cases of IFB, representing approximately 4% of all indications for emergent endoscopy [1,22]. In most circumstances, the object uneventfully passes through the gastrointestinal tract, and only 10–20% will ultimately need endoscopic removal [23]. The majority of patients will have a benign clinical course but, although rare, significant morbidity and even mortality may occur. The type of object varies according to several factors: namely, the study population and its dietary patterns, age, the prevalence of esophageal disease, the social context and a history of psychiatric disease. Food bolus impaction is more frequently described in the Western Hemisphere and affects particularly those with previous esophageal disease, the elderly and people with dementia [10,24]. Bones (particularly fish bones) dominate in Asian countries [2,3,25,26]. The ingestion of foreign bodies other than food most frequently involves children, prisoners and people with cognitive impairment or psychiatric disease.

Caustic injury is generally considered a rare event but with the potential for severe consequences. The vast majority of cases occur with children and are almost always of an accidental nature, typically involving the ingestion of smaller volumes, resulting in milder lesions, which seldom require specific treatment or follow-up. Conversely, ingestions in adults more frequently occur in a deliberate attempt to self-harm. The volumes of the ingested solution tend to be larger [15,27]. In such circumstances, damage to the gastrointestinal tract is usually extensive, and hospital admission is frequently necessary.

In this cohort, 41 of 44 patients (93%) admitted for IFB underwent upper endoscopy. The foreign body was retrieved in almost 59% of these patients, which is in line with other reports [3,10]. Fish bones were the most frequently retrieved foreign body, followed by food boluses. Of all the IFB admissions, only 11% (*n* = 5) occurred after voluntary ingestion, which is in agreement with previous data [4]. A total of four out of five of these patients had a previous diagnosis of psychiatric disease. However, events of IC were more frequently intentional (77%), and most of these patients (82%) had a previous diagnosis of psychiatric disease. This number is higher than those previously published by others [27,28,29]. This disparity may be explained by differences in the study populations, as our study focused on hospitalized patients, with greater overall severity, which more frequently occur after intentional ingestion.

In this retrospective cohort, we found that the CRP values may have helped to predict the clinical outcomes in the IFB patients. In this group of patients, the CRP values correlated to longer hospital stays. We postulate that this relationship may have been mediated by complications associated with systemic inflammation, such as the development of periesophageal collections and esophageal perforation. In fact, these patients had higher CRP levels than the patients without such complications. In contrast, an association between the CRP levels and LS was not seen in the IC patients.

Endoscopy was performed in more than 90% of the patients in both groups. The majority of patients underwent an upper endoscopy less than 24 h after presentation. The timing of the endoscopy had no impact on the LS or the other inpatient measures of outcome. This contrasts with the findings of several reports that describe an association between the delay in endoscopy or prolonged foreign body retention and the incidence of complications [4,9,11,25,30]. Yuan and coworkers have reported an increase of four times the risk of complications for those submitted to endoscopy >24 h after IFB [4]. Also, delay in the performance of the endoscopy has been shown to decrease the probability of finding the foreign body and its successful removal [9,10]. Similarly, in IC patients, prolonged time between presentation and endoscopy has been shown to increase the risk of poor clinical outcomes, including prolonged hospitalization and in-hospital death [31].

Several studies have evaluated the usefulness of the Zargar classification for predicting outcomes in patients with caustic injury. Cheng et al. evaluated the relationship between the Zargar classification and several outcome measures and reported significant associations between this classification and the length of hospitalization, the need for ICU treatment, systemic complications, such as respiratory failure and gastrointestinal complications, as well as late complications, such as the development of strictures [16]. Moreover, it has been shown that this classification may help to predict the risk of death after IC [32]. In this work, there was no significant association between the Zargar classification and the primary outcome: the length of stay. Two reasons may have contributed to this: first, the number of IC patients with descriptions of this classification was small, which may have hampered our ability to find a significant correlation; second, patients with more severe lesions (grade 3B) were relatively underrepresented in our cohort when compared to other studies in which such correlations were found [16]. Nevertheless, moderate-to-severe caustic lesions were associated with higher requirements of intensive care treatment, the use of antibiotics and the incidence of respiratory complications.

A debate exists regarding the roles of endoscopy and CT scans after IC. The CT scan provides an accurate assessment of the transmural extension of necrosis compared to endoscopy, and it has demonstrated significant predictive power for the development of complications [14]. In our study, no association was found between the findings of the CT scans and outcomes in the IC patients. Furthermore, the CT scan severity index, as proposed by Ryu et al. [21], did not correlate with the length of hospitalization or any other inpatient outcome measures. Again, the small number of IC cases for which a CT scan was performed may have limited our ability to find significant associations in this subset of patients. However, CT findings of periesophageal fluid collections and perforation after IFB significantly correlated with an increased LS.

Over 45% of the patients admitted for IFB had endoscopic or tomographic signs of perforation. This figure is much higher than those reported by other authors [24,25]. This may reflect the significant proportion of these patients that had simultaneous endoscopy and CT scan, which may have increased the yield of detection of these lesions. Ultimately, over a quarter of the IFB patients underwent surgical treatment for complications. The reason for surgery was perforation in 10 out of 12 patients. Only four patients with caustic injury were submitted to surgery for complications. This value is lower than others previously reported [27].

This work has merit in being one of the first to describe and compare the predictive factors of the outcomes of patients hospitalized due to these common etiologies of non-hemorrhagic endoscopic emergencies. However, our study has certain limitations. First, it is of a retrospective design and includes a small number of patients, which may have limited our ability to find significant correlations. Also, the information was recovered from patients’ clinical records, which are susceptible to incompletion.

## 5. Conclusions

Different factors are correlated with inpatient outcomes for patients admitted due to IFB or IC. For the first group, the levels of CRP upon admission are predictive of longer hospitalization. Also, evidence of perforation on endoscopy or CT scan are predictive of worse inpatient outcomes. However, for those admitted for IC, the severity of the esophageal lesions on endoscopy or CT scan had no implications on the duration of the hospital stay. Nevertheless, the Zargar classification may prove useful in predicting in-hospital complications, such as the need for ICU treatment or infectious complications.

## Figures and Tables

**Table 1:** Demographic, clinical and laboratory data.

	IFB (*n* = 44)	IC (*n* = 22)	Total (*n* = 66)	*p*
Male, *n* (%)	28 (64%)	14 (64%)	42 (64%)	1
Age, mean (range)	56.3 (19–89)	50.4 (18–83)	54.3 (18–89)	0.248
Dementia, *n* (%)	4 (9%)	2 (9%)	6 (9%)	1
Psychiatric disease, *n* (%)	8 (18%)	18 (82%)	26 (39%)	<0.0001
CCI, median (IQR)	2.0 (0.3–5.0)	1.0 (0.0–4.0)	2.0 (0.0–4.3)	0.109
Esophageal motor disturbances, *n* (%)	4 (9%)	0	4 (6%)	0.293
Motivation				
Intentional, *n* (%)	5 (11%)	17 (77%)	22 (33%)	<0.0001
Symptoms				
Asymptomatic, *n* (%)	4 (10%)	2 (12%)	6 (11%)	1
Dysphagia or odynophagia, *n* (%)	29 (73%)	12 (71%)	41 (72%)	1
Abdominal or thoracic pain, *n* (%)	17 (43%)	11 (65%)	28 (49%)	0.155
Hypersalivation, *n* (%)	13 (33%)	9 (53%)	22 (39%)	0.234
Vomit, *n* (%)	10 (25%)	6 (35%)	16 (28%)	0.523
Hematamesis, *n* (%)	5 (13%)	6 (35%)	11 (19%)	0.068
Dyspnea, *n* (%)	6 (15%)	5 (29%)	11 (19%)	0.275
Melena or rectal bleeding, *n* (%)	6 (15%)	1 (6%)	7 (12%)	0.662
Lab values				
Hb (g/dL), mean ± SD	12.1 ± 3.0	13.5 ± 2.9	12.5 ± 3.1	0.086
WBC (×10^9^/L), mean ± SD	10.3 ± 3.9	11.1 ± 3.1	10.5 ± 3.7	0.407
CRP (mg/L), median (IQR)	19.1 (7.5–99.5)	10.4 (5.4–32.8)	16.0 (6.0–64.2)	0.222

IFB—ingestion of foreign bodies; IC—ingestion of caustics; CCI—Charlson comorbidity index; IQR—interquartile range; Hb—hemoglobin; WBC—white blood cell count; CRP—C-reactive protein; SD—standard deviation.

**Table 2:** Upper-endoscopy findings.

	IFB (*n* = 41)	IC (*n* = 20)	Total (*n* = 61)	*p*
Endoscopy performance				
Endoscopy, *n* (%)	41 (93%)	20 (91%)	61 (92%)	1
Timing for upper endoscopy				
<24 h, *n* (%)	33 (81%)	14 (70%)	47 (77%)	0.517
Location of lesions				
Oropharynx, *n* (%)	3 (7%)	2 (11%)	5 (8%)	
Upper esophagus, *n* (%)	11 (26%)	2 (5%)	13 (22%)	
Mid-esophagus, *n* (%)	18 (44%)	2 (11%)	20 (34%)	
Lower esophagus, *n* (%)	9 (22%)	1 (6%)	10 (17%)	
Pan-esophageal, *n* (%)	0	11 (61%)	11 (19%)	
Foreign body ingestion lesions				
No lesions, *n* (%)	12 (29%)			
Mucosal ulceration, *n* (%)	17 (42%)			
Perforation, *n* (%)	12 (29%)			
Zargar classification of caustic lesions				
0, *n* (%)		1 (5%)		
1, *n* (%)		6 (32%)		
2A, *n* (%)		2 (11%)		
2B, *n* (%)		3 (16%)		
3A, *n* (%)		4 (21%)		
3B, *n* (%)		3 (16%)		

Outcomes: IFB—ingestion of foreign bodies; IC—ingestion of caustics.

**Table 3:** CT scan findings.

	IFB (*n* = 34)	IC (*n* = 14)	Total (*n* = 48)	*p*
CT performance				
CT, *n* (%)	34 (77%)	14 (64%)	48 (73%)	0.257
CT Findings				
No alterations, *n* (%)	11 (32%)	3 (21%)	14 (29%)	0.510
Wall edema, *n* (%)	9 (27%)	3 (21%)	12 (25%)	1
Edema and soft-tissue involvement, *n* (%)	1 (3%)	4 (29%)	5 (10%)	0.021
Periesophageal fluid collection, *n* (%)	7 (21%)	4 (29%)	11 (23%)	0.708
Perforation, *n* (%)	18 (53%)	2 (14%)	20 (42%)	0.023
CT scan severity index				
Grade 1, *n* (%)		3 (21%)		
Grade 2, *n* (%)		3 (21%)		
Grade 3, *n* (%)		4 (29%)		
Grade 4, *n* (%)		4 (29%)		

IFB—ingestion of foreign bodies; IC—ingestion of caustics; CT—computerized tomography.

**Table 4:** Outcomes and clinical complications.

	IFB (*n* = 44)	IC (*n* = 22)	Total (*n* = 66)	*p*
Complications				
LS, median (IQR)	6.0 (3.0–14.3)	9.0 (5.0–20.0)	7.0 (4.0–15.0)	0.066
ICU, *n* (%)	14 (32%)	10 (46%)	24 (36%)	0.293
Antibiotics, *n* (%)	27 (61%)	13 (59%)	40 (61%)	1
Vasopressor support, *n* (%)	2 (5%)	1 (5%)	3 (5%)	1
Respiratory failure, *n* (%)	2 (5%)	4 (18%)	6 (9%)	0.090
Pneumonia, *n* (%)	7 (16%)	6 (27%)	13 (20%)	0.331
Bleeding with need for transfusion, *n* (%)	12 (27%)	2 (9%)	14 (21%)	0.117
Perforation, *n* (%)	20 (46%)	2 (9%)	22 (33%)	0.005
Peritonitis, *n* (%)	0	0	0	-
Mediastinitis, *n* (%)	9 (21%)	4 (18%)	13 (20%)	1
Mortality, *n* (%)	0	0	0	-
Treatment of complications				0.283
Medical, *n* (%)	29 (66%)	18 (82%)	47 (71%)	
Endoscopic stent, *n* (%)	3 (7%)	0	3 (5%)	
Surgery, *n* (%)	12 (27%)	4 (18%)	16 (24%)	

IFB—ingestion of foreign bodies; IC—ingestion of caustics; LS—length of stay; ICU—intensive care unit; IQR—interquartile range.

**Table 5:** Correlations of clinical, laboratory, endoscopic and imaging variables with length of stay.

	IFB (*n* = 44)	IC (*n* = 22)	Total (*n* = 66)
Demographic variables			
Age, *ρ* (*p*)	0.060 (0.701)	0.285 (0.198)	0.108 (0.389)
CCI, *ρ* (*p*)	−0.273 (0.073)	0.069 (0.759)	−0.201 (0.106)
Laboratory variables			
Hb, *ρ* (*p*)	−0.013 (0.933)	−0.053 (0.821)	0.004 (0.974)
WBC, *ρ* (*p*)	0.095 (0.544)	0.215 (0.348)	0.182 (0.149)
CRP, *ρ* (*p*)	0.542 (<0.01) **	0.180 (0.435)	0.368 (0.03) *
Endoscopic variables			
Endoscopy timing, *ρ* (*p*)	0.271 (0.087)	0.615 (0.120)	0.245 (0.057)
FBI lesions, *ρ* (*p*)	0.425 (0.006) **		-
Zargar classification, *ρ* (*p*)	-	0.221 (0.362)	-
CT scan variables			
Periesophageal fluid collection, *ρ* (*p*)	0.361 (0.036) *	0.413 (0.142)	0.395 (0.005) **
Perforation, *ρ* (*p*)	0.628 (<0.01) **	0.406 (0.149)	0.424 (0.003) **
CT scan severity index, *ρ* (*p*)	-	0.360 (0.207)	-

IFB—ingestion of foreign bodies; IC—ingestion of caustics; CCI—Charlson comorbidity index; Hb—hemoglobin; WBC—white blood cell count; CRP—C-reactive protein; CT—computed tomography; *ρ*—Spearman’s *ρ*. * *p* < 0.05; ** *p* < 0.01.

## Data Availability

Data are available upon reasonable request.
